# Supplementary material for: Genome-Wide Survey and Expression Analyses of Hexokinase Family in Poplar (Populus trichocarpa)
Source: Plants (Basel). 2022 Aug 3;11(15):2025. doi: 10.3390/plants11152025 (PMC9370503; doi:10.3390/plants11152025)
Supplement: Supplementary file 1 [file plants-11-02025-s001.zip › plants-1849713-supplementary.pdf]

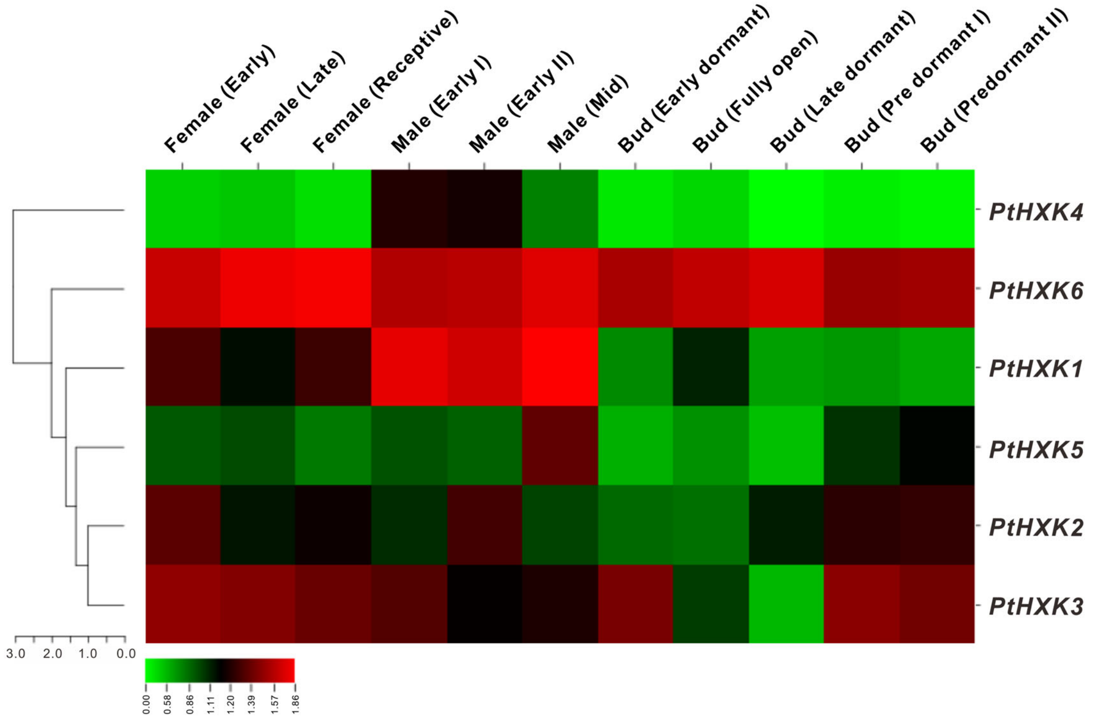

Figure S1. Transcriptomic profiles of *PtHXKs* in reproductive tissues. The heatmap shows the transcript in various reproductive tissues of *P. trichocarpa*. The RNA-seq data were given in the Log10 of the fragments per kilo-base per million reads (FPKM) expression values.

Table S1. List of HXK candidate genes identified in *P. trichocarpa*.

Gene ID (v3.1)	Name	DNA (bp)	ORF (bp)	Size (a.a)	MW (kDa)	PI	TP	Target	Identity*
Potri.001G190400	*PtHXK1*	4989	1485	494	53.63	5.70	1-21	Mito	87.0/AtHXK1
Potri.001G254800	*PtHXK2*	5709	1527	508	54.97	6.50	1-20	Mito	85.7/AtHKL1
Potri.005G238600	*PtHXK3*	5557	1488	495	53.09	5.65	1-59	Chl	79.2/AtHXK3
Potri.007G009300	*PtHXK4*	3611	1473	490	53.57	6.06	1-23	Mito	76.4/AtHKL3
Potri.009G050000	*PtHXK5*	5679	1527	508	54.91	6.54	1-20	Mito	85.3/AtHKL1
Potri.018G088300	*PtHXK6*	6125	1497	498	53.82	5.63	1-19	Mito	90.1/AtHXK1

* The identity percentage to *Arabidopsis* HXKs. Mito, mitochondrion; Chl, chloroplast.TP, transit peptide.

Table S2. List of primers used for qRT-PCR analyses.

Primer Name	Sequence (5′-3′)	Access number/Gene
qFW187	GCGTCAGCAATTAATGAGGAGT	Potri.001G190400/*PtHXK1*
qRV187	GATCGAGTTTGCTCGCTCTC	
qFW188	CTACAGCGTGGATGGCGTAA	Potri.001G254800/*PtHXK2*
qRV188	CATTGAGGTACGAGGACCGT	
qFW189	TCCTGGCTTATATGCACCCT	Potri.005G238600/*PtHXK3*
qRV189	AAACCTTAGCACTTGATTGAGC	
qFW190	AAGATGGCCCAGGAAACAGC	Potri.007G009300/*PtHXK4*
qRV190	ATTTGTGATCCCAAGATTTTCCTTC	
qFW191	AGTACCGTCCAGTTCGCTTT	Potri.009G050000/*PtHXK5*
qRV191	TTCAGCGGCGAGAGACAAAC	
qFW192	TGGGACCCCTATTGGGAAGT	Potri.018G088300/*PtHXK6*
qRV192	TTGTTCCGCCAAGGTCCAAT	
UBIC_qF2	TTGCTTGAGGAACTTGAAC	Potri.006G205700.7/*PtUBIC*
UBIC_qR2	GTGAGGACCGATTACAGT	
β-Actin7_qF	GACCTTCAATGTGCCTGCAA	Potri.019G006700.1/*PtβActin*
β-Actin7_qR	ACCATCACCAGAATCCAGCA	
EF1α_qF	TCCGTCTTCCACTTCAGGATGTCT	Potri.006G130900.1/*PtEF1α*
EF1α_qR	GTCACGACCATACCAGGCTTCAG	

Table S3. List of gene ID and name used in Phylogenetic tree.

Species	Name	Gene ID	Species	Name	Gene ID	Species	Name	Gene ID
Arabidopsis	*AtHKL1*	AT1G50460	*Gossypium*	*GrHXK3*	Gorai.009G137700	*Phyllostachys*	*PeHXK9*	PH02Gene15132.t1
thaliana	*AtHKL2*	AT3G20040	*raimondii*	*GrHXK4*	Gorai.010G004700	*edulis*	*PeHXK10*	PH02Gene40020.t1
	AtHKL3	AT4G37840		*GrHXK5*	Gorai.010G077400	*Physcomitrella*	*PpHXK1*	AY260967
	AtHXK1	AT4G29130		*GrHXK6*	Gorai.011G070700	*Patens*	*PpHXK2*	XM_001784578.1
	AtHXK2	AT2G19860		*GrHXK7*	Gorai.013G251900		*PpHXK3*	XM_001784282.1
	AtHXK3	AT1G47840		*GrHXK8*	Gorai.013G230400		*PpHXK4*	XM_001760896.1
Brachypodium	*BradiHXK1*	Bradi2g05670.2	*Manihot*	*MeHXK1*	Manes.18G014000		*PpHXK5*	XM_001766381.1
distachyon	*BradiHXK2*	Bradi2g18877.1	*esculenta*	*MeHXK2*	Manes.16G109200		*PpHXK6*	XM_001762899
	BradiHXK3	Bradi2g19400.4		*MeHXK3*	Manes.06G161400		*PpHXK7*	XM_001754096
	BradiHXK4	Bradi2g27150.1		*MeHXK4*	Manes.14G005400		*PpHXK8*	XM_001752177
	BradiHXK5	Bradi2g33380.1		*MeHXK5*	Manes.04G134700		*PpHXK9*	XM_001770125
	BradiHXK6	Bradi2g48547.1		*MeHXK6*	Manes.03G026700		*PpHXK10*	XM_001776713
	BradiHXK7	Bradi2g60450.1		*MeHXK7*	Manes.12G119601		*PpHXK11*	XM_001779426
	BradiHXK8	Bradi4g43820.1	*Medicago*	*MedtrHXK1*	Medtr1g025140	*Populus*	*PtHXK1*	Potri.001G190400
	BradiHXK9	Bradi5g01836.1	*truncatula*	*MedtrHXK2*	Medtr4g097900	*trichocarpa*	*PtHXK2*	Potri.001G254800
Eucalyptus	*EucgrHXK1*	Eucgr.B03711		*MedtrHXK3*	Medtr5g009000		*PtHXK3*	Potri.005G238600
grandis	*EucgrHXK2*	Eucgr.C00559		*MedtrHXK4*	Medtr6g088795		*PtHXK4*	Potri.007G009300
	EucgrHXK3	Eucgr.C00569		*MedtrHXK5*	Medtr8g014530		*PtHXK5*	Potri.009G050000
	EucgrHXK4	Eucgr.C00975		*MedtrHXK6*	Medtr8g102460		*PtHXK6*	Potri.018G088300
	EucgrHXK5	Eucgr.C00983	*Nicotiana*	*NtHXK1a*	LOC107807808	*Solanum*	*SlHXK1*	Solyc03g121070.2.1
	EucgrHXK6	Eucgr.C03728	*tabacum*	*NtHXK2*	LOC107790581	*lycopersicum*	*SlHXK2*	Solyc06g066440.2.1
	EucgrHXK7	Eucgr.F01647		*NtHXK3*	LOC107766493		*SlHXK3*	Solyc12g008510.1.1
	EucgrHXK8	Eucgr.J00734		*NtHXK4a*	LOC107810082		*SlHXK4*	Solyc04g081400.2.1
Ginkgo biloba	*GbHXK1*	Gb_04841		*NtHXK4b*	LOC107770541		*SlHXK5*	Solyc11g065220.1.1
	GbHXK2	Gb_15682		*NtHXK6*	LOC107788280		*SlHXK6*	Solyc02g091830.2.1
	GbHXK3	Gb_18025	*Oryza sativa*	*OsHXK1*	LOC_Os07g26540	*Selaginella*	*SmHXK1*	231902
	GbHXK4	Gb_29423		*OsHXK2*	LOC_Os05g45590	*moellendorffii*	*SmHXK2*	441916
	GbHXK5	Gb_29587		*OsHXK3*	LOC_Os01g71320.1		*SmHXK3*	150326
	GbHXK6	Gb_38982		*OsHXK4*	LOC_Os07g09890		*SmHXK5*	117919
Glycine max	*GlymaHXK1*	Glyma.01G007200		*OsHXK5*	LOC_Os05g44760	*Sorghum bicolor*	*SobicHXK1*	Sobic.003G035500

	GlymaHXK2	Glyma.01G007300		*OsHXK6*	LOC_Os01g53930		*SobicHXK2*	Sobic.003G280400
	GlymaHXK3	Glyma.01G226900		*OsHXK7*	LOC_Os05g09500		*SobicHXK3*	Sobic.003G291800
	GlymaHXK4	Glyma.05G110500		*OsHXK8*	LOC_Os01g09460		*SobicHXK4*	Sobic.003G421201
	GlymaHXK5	Glyma.05G226600		*OsHXK9*	LOC_Os01g52450		*SobicHXK5*	Sobic.009G069800
	GlymaHXK6	Glyma.07G124500		*OsHXK10*	LOC_Os05g31110		*SobicHXK6*	Sobic.009G119100
	GlymaHXK7	Glyma.08G033300	*Phyllostachys*	*PeHXK1*	PH02Gene48829.t1		*SobicHXK7*	Sobic.009G203500
	GlymaHXK8	Glyma.08G200600	*edulis*	*PeHXK2*	PH02Gene46796.t1	*Zea mays*	*ZmHXK1*	LOC100192075
	GlymaHXK9	Glyma.11G015800		*PeHXK3a*	PH02Gene41019.t1		*ZmHXK2*	LOC100170246
	GlymaHXK10	Glyma.11G095600		*PeHXK3b*	PH02Gene29892.t1		*ZmHXK3*	LOC542510
	GlymaHXK11	Glyma.12G021700		*PeHXK4*	PH02Gene31153.t1		*ZmHXK4*	LOC103650768
	GlymaHXK12	Glyma.14G218800		*PeHXK5a*	PH02Gene24831.t1		*ZmHXK5*	LOC103651223
	GlymaHXK13	Glyma.17G156200		*PeHXK5b*	PH02Gene08025.t1		*ZmHXK6*	LOC100279587
	GlymaHXK14	Glyma.17G257800		*PeHXK6*	PH02Gene46133.t1		*ZmHXK7*	LOC100283735
Gossypium	*GrHXK1*	Gorai.006G096800		*PeHXK7*	PH02Gene45438.t1		*ZmHXK8*	LOC100285932
raimondii	*GrHXK2*	Gorai.007G057600		*PeHXK8*	PH02Gene06290.t1		*ZmHXK9*	LOC103636300
